# Shame and Guilt Proneness as Mediators of PTSD/DSO Symptoms in Young Adults

**DOI:** 10.1002/cpp.70131

**Published:** 2025-07-29

**Authors:** Osmano Oasi, Mark Shevlin, Antonio Lasalvia, Chiara Bonetto, Doriana Cristofalo, Giulia Marzocco, Camilla Somma, Marco Toscano, Thanos Karatzias, Cesare Cavalera

**Affiliations:** ^1^ Department of Psychology Università Cattolica del Sacro Cuore Milan Italy; ^2^ School of Psychology Ulster University Derry Northern Ireland UK; ^3^ Department of Neuroscience, Biomedicine and Movement Sciences University of Verona Verona Italy; ^4^ Centre for Medical Sciences (CISMED), Chair of Psychiatry University of Trento Trento Italy; ^5^ ASST Rhodense Azienda Socio‐Sanitaria Territoriale (ASST) Rhodense Garbagnate Milanese Italy; ^6^ School of Health and Social Care Edinburgh Napier University Edinburgh Scotland UK

## Abstract

The aim of this study was to investigate the relationships between trauma exposure, shame and guilt proneness and the development of PTSD and Disturbances in Self‐Organisation (DSO) symptoms in young adults. Specifically, we hypothesised that trauma exposure would be positively correlated with PTSD and DSO symptoms and that shame and guilt would mediate this relationship. A total of 160 young adults participated in this study. Three models were tested: (1) a model with direct effects from trauma exposure to PTSD and DSO, (2) an indirect effects model where the direct paths were constrained and (3) a full model with both direct and indirect effects. Shame and guilt proneness showed a strong correlation with PTSD and DSO. Direct effects revealed that trauma exposure predicted PTSD, DSO, guilt and shame proneness. Guilt had a strong effect on PTSD, while shame had the strongest effect on DSO. Indirect effects showed that trauma exposure significantly predicted both PTSD and DSO through heightened guilt and shame. The strongest indirect relationships were trauma exposure to PTSD via guilt and trauma exposure to DSO via shame. This study demonstrates that trauma exposure is associated with heightened levels of shame and guilt proneness, which, in turn, predict greater severity of PTSD and DSO symptoms. These findings suggest that emotional regulation, particularly in relation to shame and guilt proneness, should be targeted in interventions for trauma‐related disorders. Future research should further explore the role of these emotions in the development of complex PTSD.

## Summary

- Clinicians should routinely assess guilt and shame proneness in young adults with trauma histories, as these emotional tendencies significantly predict the severity of both PTSD and DSO symptoms.
- Because guilt proneness appears to be more closely associated with PTSD and shame proneness with DSO, tailoring treatments to target these specific emotions (e.g., self‐compassion therapy for shame and cognitive restructuring for guilt) may enhance therapeutic effectiveness.
- Guilt and shame proneness may reflect long‐standing emotional processing styles shaped by repeated trauma. These patterns are especially visible in young adulthood and may require longer term or developmental‐informed care.
- Integrating emotion regulation–focused protocols such as ESTAIR or EMDR can be particularly beneficial for patients with complex trauma profiles, helping reduce both PTSD and DSO symptoms through improved emotional processing.

## 1 Introduction

The relationship between trauma exposure and its impact on mental health outcomes is well established. The presence of multiple stressful events can lead to a sense of losing control and an increased feeling of helplessness. Whether these events threaten personal safety or the integrity of the self, they may contribute to the development of symptoms of PTSD and Disturbances in Self‐Organisation (DSO) (Brewin et al. 2017; Hyland et al. 2023). The DSO symptoms can be defined in terms of how the person thinks about oneself and relates to others and include ‘affect dysregulation’, ‘negative self‐concept’ and ‘disturbances in relationship’ (Cloitre et al. 2018). While PTSD involves symptoms such as re‐experiencing the trauma, active avoidance and a persistent sense of threat, DSO symptoms reflect more pervasive and enduring changes in emotional regulation, self‐identity and interpersonal functioning, typically resulting from prolonged or repeated traumatic events (Brewin et al. 2017; Sarr et al. 2024).

More specifically, young adults are vulnerable to psychological distress because of the numerous stressors they face, such as job and academic pressures, relationship challenges and career uncertainty (Achdut 2023; Liu and Zhang 2020). This vulnerability has been further amplified by the impact of COVID‐19 (Sun and Zhou 2023). Given the established link between trauma exposure and PTSD symptoms, recurring maladaptive experiences may also contribute to the development of elevated negative emotional responses, such as shame and guilt (Cavalera et al. 2022). These two self‐conscious emotions are closely tied to the self, yet they differ in important ways. While shame involves a global devaluation of the self (‘I am a bad person’), guilt focuses on the evaluation of a specific action (‘I did a bad thing’; Lewis 1971), and they are associated with distinct neurobiological correlates (Michl et al. 2014). Repeated traumatic experiences can contribute to the development of tendencies towards shame and guilt. These emotions can become deeply ingrained in a person's sense of self, often leading to shame proneness or guilt proneness. (Tangney et al. 2011). Shame proneness may emerge when the meaning of recursive stressful events aligns with pre‐existing shame schemas, which have profound implications for the self and/or others, or when there is a disruption of a positive self‐identity. On the other hand, guilt proneness tends to arise when the meaning of the traumatic event reflects a deviation from behavioural norms and/or a sense of responsibility for causing harm to others. When self‐criticism and hyper‐responsibility become excessively elevated, guilt proneness may become maladaptive and impair functioning (Shi et al. 2021).

The recursive devaluation of personal worth and behaviour that characterises both shame and guilt proneness may play a crucial role in the development of PTSD and DSO symptomatology (Cunningham et al. 2017, 2019). This topic warrants further investigation, as both guilt and shame can interfere with various cognitive and emotional domains, such as identity recognition, emotional regulation and interpersonal functioning, which are fundamental to the development of DSO (Quiroga‐Garza and Cavalera 2024; Volpato et al. 2022). Nonetheless, guilt proneness seems to be specifically linked to PTSD symptoms, as maladaptive hyper‐responsibility may contribute to intrusive memories and reinforce avoidance behaviours related to wrong actions, such as surviving when others did not or believing one should have acted differently (Cândea and Szentagotai‐Tătar 2018). On the other hand, shame proneness appears to be more closely associated with DSO as it reflects a pervasive sense of being damaged and it contributes to feelings of worthlessness and long‐term impairments in self‐esteem and identity (Cavalera et al. 2023). This view is consistent with a recent study by Békés et al. (2023) that found that shame was a significant mediator of the relationship between trauma exposure and DSO in an adult inpatient psychiatric sample. These initial results are promising, but further studies are crucial to deepen our understanding of how guilt and shame may contribute to PTSD and DSO, considering also non‐clinical populations.

On the basis of the extant research, it was hypothesised that there would be significant positive relationships among measures of trauma exposure, shame and guilt proneness and PTSD and DSO symptoms. However, since the introduction of complex PTSD to the ICD‐11 (WHO 2018), there has been no research that has examined the differential associations between PTSD, DSO symptoms and shame and guilt proneness. Therefore, the primary aim of this study was to assess the relationship between these variables and test a model that proposes shame and guilt proneness as mediators of the trauma–PTSD/DSO association in a young‐adult sample consisting of patients and non‐patients matched controls. By including both outpatients and matched controls, this study allows for valuable insights into the role of trauma and its psychological correlates on individuals with and without a history of mental treatment. This approach also makes it possible to examine whether participant status, that is, having or not having a history of mental healthcare, influences the associations between the key variables of interest. Specifically, it was hypothesised that (1) trauma exposure would be positively related to PTSD and DSO symptoms; (2) trauma exposure would be positively related to shame and guilt proneness; (3) shame and guilt proneness would be positively related to PTSD and DSO symptoms, with shame particularly contributing more to the presence of DSO symptoms, while guilt would be more strongly associated with PTSD symptoms; and (4) in an overall model, there would be significant direct and indirect (via shame and guilt) effects between trauma exposure and PTSD and DSO symptoms, with shame and guilt proneness potentially acting as mediators in this relationship (while controlling for patient/control status).

## 2 Methods

### 2.1 Participants and Procedures

A total of 160 young adults (age range 18–36, M = 25.89, SD = 5.26) were recruited. Of these, 80 were outpatients from two mental health departments located in northern Italy: Verona Academic Hospital Trust and Azienda Socio‐Sanitaria Territoriale Rhodense in Garbagnate Milanese. These patients were selected by their referring psychiatrists. Inclusion criteria for outpatients were being aged between 18 and 36 years and currently under mental health treatment. The remaining 80 participants were control subjects from northern Italy, included via snowball sampling. They were selected only if they had never received mental healthcare and could be matched to outpatients by age and gender. Participation was voluntary and anonymous. Data were collected in the index period from April 2022 to September 2024. All participants provided written informed consent before the start of assessments. We conducted a power analysis to estimate the required sample size given the following parameters: target power = 0.80, two parallel mediators and a (conservative) specification of correlations among all the variables being *r* = 0.40. The required sample size based on Monte Carlo methods (Schoemann et al. 2017) was *N* = 141, and there was over‐recruitment in case of significant missing data.

### 2.2 Transparency and Openness

Data were analysed using IBM SPSS Version 23.0, and the latent variable models were specified and estimated using Mplus 8.0. Data are available upon reasonable request from the corresponding authors. The study design was registered on PsyArXiv. Data for the outpatient group were collected by the treating physicians, while control data were collected simultaneously, with only those matching the outpatient group in terms of gender and age being included. Ethical approval was granted by the ethical committees of the coordinating centre (Università Cattolica di Milano) and the other recruiting centres.

### 2.3 Measures

#### 2.3.1 Traumatic Exposure

Traumatic exposure was assessed using the International Trauma Exposure Measure (ITEM: Hyland et al. 2021), which is a freely available checklist measuring 21 threatening life events. Sixteen events reflect the DSM‐5 definition of trauma exposure (i.e., direct or indirect threat to life or to physical or sexual safety; e.g. ‘someone threatened your life with a weapon’). The other five events are psychologically threatening events that can be considered traumatic in line with ICD‐11 guidelines (e.g. ‘you were repeatedly neglected, ignored, rejected or isolated’). Respondents indicate if they experienced each event during three developmental periods (0–12 years, 13–18 years and older than 18 years). Scores are binary coded (No = 0, Yes = 1) and summed for each participant producing scores with a possible range of 0–21.

#### 2.3.2 ICD‐11 PTSD and CPTSD

The International Trauma Questionnaire (ITQ: Cloitre et al. 2018) was used for the assessment of ICD‐11 symptoms of PTSD and CPTSD. The ITQ is a self‐report scale measuring all diagnostic requirements for PTSD and CPTSD. Respondents identify their index trauma event and how long ago it occurred. They are then instructed to answer all questions in relation to this event. There are six items measuring PTSD symptoms across the clusters of ‘Re‐experiencing in the here and now’ (Re), ‘Avoidance’ (Av) and ‘Sense of Threat’ (SoT). The DSO symptoms are answered in terms of how the respondent typically feels, thinks about oneself and relates to others. The PTSD and DSO subscales have possible range of scores of 0–8. Three items measure functional impairment associated with these symptoms. Multiple studies with general population samples have shown that the ITQ scores possess satisfactory reliability and validity (see Redican et al. 2021 for review). The internal reliability (Cronbach's alpha) estimates of the PTSD (*α* = 0.85) and DSO (*α* = 0.90) subscales in this sample were excellent.

#### 2.3.3 PFQ‐2 Shame and Guilt Proneness

The Personal Feelings Questionnaire‐2 (PFQ‐2; Di Sarno et al. 2022; Harder and Zalma 1990; Harder and Greenwald 1999) is a 22‐item self‐report tool designed to assess proneness to shame (e.g., ‘embarrassment’ and ‘feeling ridiculous’) and proneness to maladaptive guilt (e.g., ‘regret’ and ‘intense guilt’) (Vigfusdottir et al. 2024 *)*. Participants are asked to rate how frequently they experience the feeling described in each item using a five‐point Likert scale ranging from 0 (‘Never’) to 4 (‘Continuously or almost continuously’). The measure has a two‐factor structure, with six items loading on the maladaptive guilt‐proneness factor and 10 on the shame‐proneness factor. The remaining six items serve as fillers and are not included in the scoring procedure. Higher scores indicate a greater tendency to experience the respective emotion. The measure has demonstrated acceptable internal consistency, adequate test–retest reliability and strong convergent validity. In the present study, Cronbach's alphas were 0.87 for shame proneness and 0.82 for guilt proneness.

### 2.4 Statistical Analysis

Descriptive statistics were calculated using IBM SPSS Version 23.0, and the latent variable models were specified and estimated using Mplus 8.0 (Muthén and Muthén 2017) based on maximum likelihood estimation. Group‐specific descriptive statistics for the outpatient and control group separately are reported in Table S1. The main analysis was conducted in two linked phases. First, using the combined dataset, three models were specified and tested. The aim was to test models with varying degrees of complexity and identify the simplest model that adequately explains the data. All models specified a trauma exposure variable (the summed ITEM scores, ‘Total Trauma’) predicting PTSD and DSO latent variables. Each of these latent variables was specified as being measured by the summed scores of the three symptom clusters: PTSD was measured by the scores on the Re, Av and SoT items, and DSO was measured by the scores on the ad, NSC and DR items. Model 1 was a model with only direct effects from the trauma exposure variable to the PTSD and DSO latent variables, with the indirect paths through the guilt and shame variables constrained to zero. The second model was the ‘indirect effects’ model where the direct effects from the trauma exposure variable to the PTSD and DSO latent variables constrained to zero and the indirect paths from the trauma exposure variable through the guilt and shame variables to the PTSD and DSO latent variables were estimated. The third model was the ‘full model’ that included both direct and indirect effects.

**FIGURE 1:**
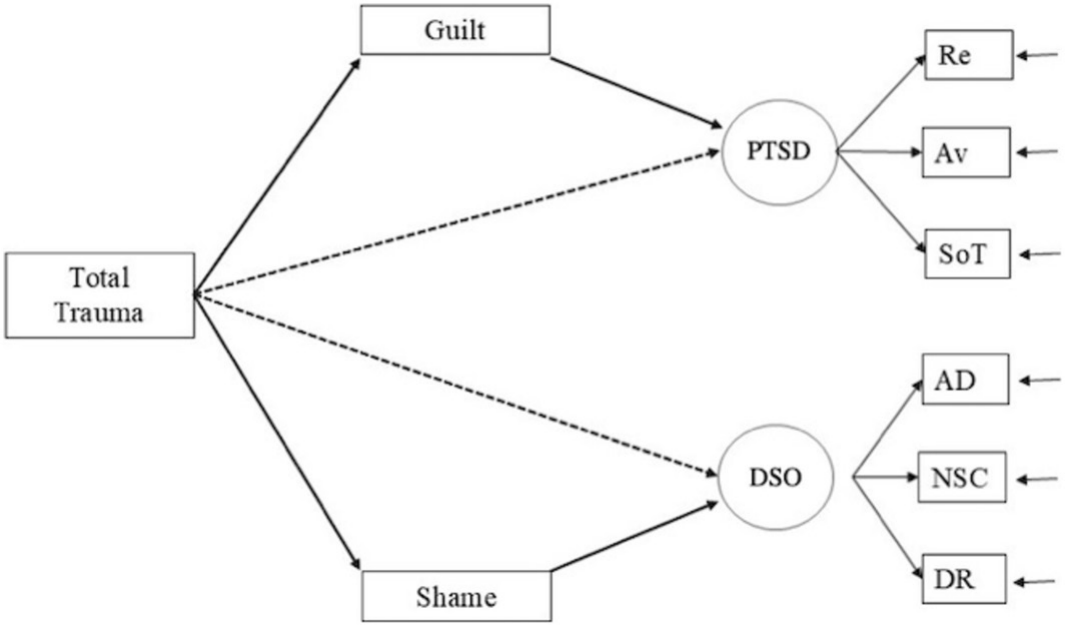
Full model with direct and indirect effects.

To account for differences between the clinical (help‐seeking outpatients) and non‐clinical (matched controls) subsamples, all path models included a binary indicator variable representing group status. This variable was specified as a control variable, such that all endogenous variables were regressed on group status, and it was allowed to covary with trauma exposure as an exogenous variable. This modelling approach ensures that parameter estimates reflect relationships among key variables in the model independent of group differences, reducing potential confounding that may arise from pooling heterogeneous samples. By statistically adjusting for clinical status, we isolate the effects of the variables without inflating associations due to group membership. Importantly, this strategy allowed us to combine data from both groups, thereby increasing the total sample size and improving statistical power: a critical consideration when testing indirect effects, which are known to require greater power than direct effects (Loeys et al. 2015). In contexts where multi‐group structural equation modelling (SEM) may not be feasible because of limited power or sample imbalance, incorporating a binary group covariate provides a parsimonious and statistically valid alternative for adjusting group‐level variance without overfitting. Overall, this approach aligns with recommended practices in SEM involving pooled samples, particularly when group moderation is not the primary focus but adjustment for group heterogeneity is essential. The overall model is illustrated in Figure 1 with the direct effects shown in dashed arrows and the indirect effects in solid arrows. The significance testing of the indirect effects was based on the principles proposed by Preacher and Hayes (2008). The statistical significance of the indirect effects was estimated using 95% bootstrapped bias‐corrected and accelerated percentile‐based confidence intervals (Efron 1987; Tibshirani and Efron 1993). Confidence intervals that do not include zero are considered to be statistically significant (*p* < 0.05) and avoid incorrect inferences about statistical significance of indirect effects. The estimates from the model should be interpreted beyond simple statistical significance, and Funder and Ozer (2019) have provided some guidance where standardised effects are described as follows: 0.10–0.20 ‘modest but meaningful’, 0.20–0.40 as ‘moderate’ and greater than 0.40 as ‘substantial’.

The following criteria were used to assess overall model fit (Hu and Bentler 1998, 1999): a non‐significant chi‐square ($\chi^{2}$), Comparative Fit Index (CFI: Bentler 1990) and Tucker Lewis Index (TLI: Tucker and Lewis 1973) values above 0.90 indicate acceptable fit; root‐mean‐square error of approximation (RMSEA: Steiger 1990) with 90% confidence intervals with values less than 0.08 indicating acceptable fit and less than 0.05 indicating ‘close’ fit. The standardised root‐mean‐square residual (SRMR: Jöreskog and Sörbom 1996) was also used with values less than 0.08 indicating acceptable fit. The Bayesian Information Criterion (BIC: Schwarz 1978) was used to compare models, with the smallest value indicating the best fitting model. Importantly, the RMSEA and the BIC both include penalties for increasing model complexity.

## 3 Results

The descriptive statistics and correlations for all the main study variables, as well as the clinical characteristics of the sample, are reported in Tables 1, S1 and 2, respectively.

**TABLE 1:** Descriptive and clinical characteristics of the outpatient (*N* = 80) and matched control (*N* = 80) groups.

	Outpatients	Matched controls	Total sample
	*n* (%)	*n* (%)	*n* (%)
Gender			
Male	35 (43.8)	35 (43.8)	70 (43.8)
Female	45 (56.2)	45 (56.2)	90 (56.2)
Age group			
18–25	43 (53.8)	43 (53.8)	86 (53.8)
25–36	37(46.2)	37(46.2)	74 (46.2)
Educational status			
Basic education	14 (19.4)	4 (5.0)	18 (11.8)
Higher education	58 (80.6)	76 (95.0)	134 (88.2)
Primary diagnosis			
Psychotic disorders	4 (5.0)		
Personality disorders	13 (16.2)		
Mood/anxiety disorders	41 (51.2)		
Trauma‐related disorders	18 (22.5)		
Other conditions	4 (5.0)		
Treatment duration *M*, (SD)	3.79 (4.30)		
Age at first contact *M*, (SD)	22.37 (3.96)		

**TABLE 2:** Descriptive statistics and correlations for all main study variables.

	1	2	3	4	5	6	7	8	9	10	11
1. Total Trauma	1.00										
2. PFQ Shame	0.287**	1.00									
3. PFQ Guilt	0.377**	0.675**	1.00								
4. ITQ Re	0.360**	0.409**	0.489**	1.00							
5. ITQ Av	0.285**	0.518**	0.554**	0.547**	1.00						
6. ITQ SoT	0.375**	0.549**	0.487**	0.569**	0.621**	1.00					
7. ITQ PTSD Total	0.398**	0.585**	0.601**	0.806**	0.864**	0.872**	1.00				
8. ITQ ad	0.350**	0.509**	0.519**	0.403**	0.481**	0.548**	0.567**	1.00			
9. ITQ NSC	0.377**	0.623**	0.586**	0.403**	0.429**	0.454**	0.506**	0.515**	1.00		
10. ITQ DR	0.344**	0.661**	0.556**	0.511**	0.542**	0.568**	0.638**	0.559**	0.548**	1.00	
11. ITQ DSO Total	0.430**	0.723**	0.670**	0.524**	0.576**	0.621**	0.679**	0.813**	0.853**	0.830**	1.00
Mean	3.99	13.08	10.04	2.50	3.65	2.80	8.94	3.62	2.44	2.60	8.66
SD	2.707	8.247	5.796	2.123	2.624	2.640	6.280	1.982	2.479	2.003	5.388

** *p* < 0.01.

The Total Trauma variable was positively and significantly correlated with both PFQ Shame (*r* = 0.287) and Guilt (*r* = 0.377), as well as total PTSD (*r* = 0.398) and DSO scores (*r* = 0.430). Shame was highly correlated with total PTSD (*r* = 0.585) and DSO scores (*r* = 0.723), as was Guilt (PTSD *r* = 0.601 and DSO *r* = 0.670).

The model fit statistics for the three models are displayed in Table 3. The fit statistics for the ‘Direct only’ model were not acceptable; the chi‐square was high relative to the degrees of freedom, the RMSEA/SRMR was too high, and the CFI/TLI too low and the BIC was the largest for all models. The fit statistics for the ‘Indirect only’ and the ‘Direct and Indirect’ models both indicated acceptable fit according to the CFI and TLI as both were greater than 0.90. The RMSEA was lower for the ‘Direct and Indirect’ model than the ‘Indirect only’ and similarly for the SRMR. The BIC was lowest for the ‘Direct and Indirect’ model, and importantly the BIC penalises more complex models—the ‘Direct and Indirect’ is the most complex with more parameters being estimated than the other models. A chi‐square difference test was significant comparing the ‘Indirect only’ and the ‘Direct and Indirect’ models (Δ^2^ = 11.352, Δdf = 2, *p* < 0.01). The ‘Direct and Indirect’ model fitted the data well, and there was evidence that the fit was superior to the other models; therefore, this was selected as the best model.

**TABLE 3:** Model fit statistics for the alternative models of trauma stage, dissociation and complex PTSD.

Models	χ^2^	df	*p*	RMSEA 95% CI	CFI	TLI	SRMR	BIC
1. Direct only	141.139	30	< 0.001	0.152 (0.127, 0.178)	0.814	0.727	0.192	6876.122
2. Indirect only	40.977	26	0.031	0.060 (0.018, 0.094)	0.991	0.983	0.046	6782.363
3. Direct and Indirect	29.625	24	0.197	0.038 (0.000, 0.078)	0.991	0.983	0.028	6780.103

The estimates of the direct effects are reported in Table 4. Total Trauma predicted PTSD (β = 0.190) and DSO (β = 0.186), as well as Guilt (β = 0.326) and Shame (β = 0.231) with the regression coefficients being slightly larger, representing moderate effect sizes. Guilt and Shame each predicted PTSD and DSO, with the strongest effects for Guilt predicting PTSD (β = 0.305) and Shame predicting DSO (β = 0.531), representing moderate‐to‐substantial effect sizes.

**TABLE 4:** Standardised estimates of direct effects.

Path	β (SE)	*p*
From Total Trauma to		
Guilt	0.326 (0.073)	< 0.001
Shame	0.231 (0.069)	< 0.01
PTSD	0.190 (0.070)	< 0.01
DSO	0.186 (0.069)	< 0.01
From Guilt to		
PTSD	0.305 (0.099)	< 0.001
DSO	0.253 (0.091)	< 0.01
From Shame to		
PTSD	0.323 (0.106)	< 0.01
DSO	0.531 (0.091)	< 0.001

The estimates of the indirect effects are reported in Table 5. All of the indirect effects of trauma on PTSD and DSO through Guilt and Shame were significant based on the assessment of the 95% bootstrapped confidence intervals with none of the ranges including zero. These findings show that the higher exposure to trauma is associated with higher levels of PTSD and DSO symptoms through heightened levels of Guilt and Shame. The standardised estimates convey that the strongest of these relationships is that of total trauma on PTSD via Guilt (β = 0.100) and total trauma on DSO via Shame (β = 0.122). The effect sizes are moderate.

**TABLE 5:** Standardised estimates of indirect effects from Total Trauma to PTSD and DSO via Guilt and Shame.

Path	β (SE)	(95% BS CI)
Indirect effect from Total Trauma to PTSD via		
Guilt	0.100	(0.023, 0.176)
Shame	0.075	(0.007, 0.142)
Indirect effect from Total Trauma to DSO via		
Guilt	0.082	(0.014, 0.151)
Shame	0.122	(0.035, 0.210)

## 4 Discussion

The present study aimed to examine the relationships between total trauma exposure, shame and guilt proneness, and symptoms of PTSD and DSO in a sample of young adults. Our results revealed significant associations between these variables, providing crucial insight into the complex interplay between trauma, emotional experience and post‐traumatic symptoms (Hyland et al. 2023). The correlations between total trauma exposure and PTSD, DSO and shame and guilt proneness were consistent with existing literature that has demonstrated a strong relationship between trauma exposure and these psychological responses (Cunningham et al. 2019). By exploring negative self‐conscious emotions in terms of enduring personality dispositions, our study offers a novel contribution to the understanding of how trauma exposure shapes long‐term emotional vulnerabilities. Notably, this study is among the first to examine how shame and guilt proneness differentially and specifically predict the mechanisms underlying PTSD and DSO symptoms, highlighting distinct pathways through which these self‐conscious emotions contribute to post‐traumatic outcomes.

### 4.1 Direct Effects of Trauma Exposure

The results indicate that trauma exposure significantly predicted not only PTSD and DSO symptoms but also guilt and shame proneness. The regression coefficients for these relationships were moderate, with trauma exposure predicting PTSD and DSO. These findings align with previous research (Brewin et al. 2017) and evidence that trauma exposure plays a central role in the onset of PTSD and DSO symptoms. High trauma exposure can challenge core beliefs about safety and self‐worth, while also triggering chronic activation of the fight‐or‐flight responses, which contribute to the onset and maintenance of PTSD (Katz et al. 2021). DSO symptoms, also resulting from trauma exposure, often manifest as a way for individuals to cope with overwhelming trauma, disrupting their sense of self and emotional regulation in an attempt to protect from further distressing experiences (Hyland et al. 2023).

Furthermore, trauma exposure also significantly predicted guilt and shame proneness. These findings highlight the importance of emotional responses in the aftermath of recursive traumatic events. Our results underscore the long‐term emotional consequences of trauma exposure, demonstrating how such experiences can shape an individual's predispositions towards these emotions and, consequently, affect their psychological functioning over time (Cavalera et al. 2023). Given that these emotional patterns often develop gradually, the long‐term effects of trauma exposure tend to manifest clearly in young adulthood, where individuals may show a stronger tendency to interpret events through stable and coherent narratives that align with their sense of self (Liu and Zhang 2020). This recursive emotional processing bias, reinforced over time, can shape the way young adults perceive and react to everyday experiences, entrenching guilt and shame‐prone dispositions and contributing to persistent emotional distress (Shi et al. 2021).

### 4.2 Role of Guilt and Shame Proneness in Predicting PTSD and DSO

Our results indicate that guilt and shame proneness each significantly predicted PTSD and DSO symptoms, albeit in different ways. These results underscore the uniqueness of our findings, which align with the increasing recognition of negative self‐conscious emotions as core mechanisms in PTSD. Shame‐prone individuals are likely to internalise stressful experiences as evidence of their inherent worthlessness or failure, which exacerbates feelings of helplessness and deepens the sense of trauma‐related distress (Békés et al. 2023; Cândea and Szentagotai‐Tătar 2018). This internalised sense of failure may reinforce the intrusive memories and avoidance behaviours characteristic of PTSD, further entrenching these symptoms in young adults (Cunningham et al. 2019).

Guilt proneness was found to have the strongest effect on PTSD symptoms. This suggests that individuals who attribute hyper‐responsibility to themselves after a traumatic event are likely to develop core PTSD symptoms (Shi et al. 2021). This finding is consistent with cognitive models of PTSD, which propose that maladaptive cognitions, such as self‐blame, contribute to the persistence of PTSD symptoms (Békés et al. 2023). The strong association between guilt proneness and these symptoms suggests that feelings of excessive responsibility and self‐blame, hallmarks of maladaptive guilt proneness, may act as maintaining factors in PTSD pathology.

Regarding DSO symptoms, shame proneness was found to have the strongest effect. This finding aligns with existing literature, as shame disrupts an individual's sense of self‐worth and coherence, leading to identity fragmentation (Békés et al. 2023). Shame‐based beliefs can lead to a defective sense of self and disrupt the intact sense of personal identity, which contributes to the emotional dysregulation and relational difficulties central to DSO (Hyland et al. 2023). Moreover, shame proneness is closely linked to chronic emotional dysregulation, a central feature of DSO. Individuals prone to shame may experience intense, prolonged affective states (e.g., self‐loathing and humiliation) that they struggle to modulate. This can lead to maladaptive coping strategies such as dissociation, withdrawal or submissive interpersonal behaviour (Cândea and Szentagotai‐Tătar 2018).

Differently, guilt proneness may contribute to DSO symptoms by hindering adaptive trauma processing (Tignor and Colvin 2019). The heightened sense of responsibility and chronic self‐blame associated with guilt may impair the integration of traumatic experiences into a coherent self‐narrative, thereby contributing to DSO symptoms. However, it is the pervasive negative self‐evaluation and identity‐related distress characteristic of shame proneness that seem to more directly underpin the core features of DSO, thereby emerging as a stronger and more consistent predictor.

### 4.3 Indirect Effects of Trauma Exposure via Guilt and Shame Proneness

As demonstrated in our results, guilt and shame proneness also played significant roles in mediating the effects of trauma exposure on PTSD and DSO symptoms. These findings suggest that trauma not only directly affects post‐traumatic symptoms but also does so through emotional processes (Hyland et al. 2023). Specifically, trauma exposure can lead to heightened levels of internalised guilt and shame, which in turn increase the severity of PTSD and DSO symptoms (Karatzias et al. 2018; Shi et al. 2021).

The indirect effects of trauma on PTSD and DSO via guilt and shame proneness further underscore the fact that individuals exposed to traumatic events often interpret their experiences through lenses of self‐blame and maladaptive hyper‐responsibility, which significantly influence how they process reality, manifesting as PTSD and DSO symptoms (Cândea and Szentagotai‐Tătar 2018). These negative emotional tendencies may have developed during childhood and been reinforced throughout adolescence in response to repeated traumatic experiences (Békés et al. 2023). As a result, they can become particularly evident in young adulthood, a period that serves as a culmination of the life experiences up until that point, where the long‐term effects of recurrent trauma exposure become apparent.

The relationship between trauma exposure, guilt and shame proneness and PTSD and DSO symptoms could be particularly relevant for individuals with pre‐existing emotional vulnerabilities, such as those with personality disorders or anxiety and mood disorders who represent the majority of the outpatients involved. Specifically, individuals with mood and anxiety disorders may demonstrate heightened susceptibility to developing PTSD symptoms because of their emotional dysregulation and difficulties in processing traumatic experiences. On the other hand, individuals with personality disorders may be more prone to experiencing DSO symptoms, given their challenges with self‐identity and interpersonal functioning.

It is important to note, however, that our model also holds true for non‐clinical participants, indicating that these relationships are not exclusive to individuals with a history of mental healthcare. This suggests that trauma‐related emotional mechanisms, such as guilt and shame proneness, have broader relevance, even in individuals who do not have a history of mental care. This further emphasises the importance of considering these emotional tendencies in both clinical and non‐clinical contexts, as they may contribute to trauma‐related distress and symptomatology across a wide range of individuals.

## 5 Conclusion

The current results emphasise the significant role of emotional experiences in mediating the effects of trauma, which may be important for understanding the mechanisms behind trauma‐related psychopathology. This study highlights the significant role of guilt and shame proneness in the development and maintenance of trauma‐related symptoms, underscoring the complexity of emotional responses following trauma exposure. The associations observed between trauma, guilt, shame and PTSD/DSO suggest that trauma‐exposed individuals with elevated levels of guilt and shame proneness are at increased risk for more severe psychological distress (Shi et al. 2021). In particular, shame proneness was found to play a greater role in predicting DSO symptoms, while guilt proneness was more strongly associated with the prediction of PTSD symptoms. This may be particularly true in light of the psychological implications of the COVID‐19 pandemic, which exacerbated trauma exposure and emotional dysregulation in many young adults (Cavalera et al. 2023). These findings represent a novel contribution to the field, as they provide empirical evidence that not just single‐state emotions but different self‐conscious emotional tendencies uniquely predict PTSD and DSO in young adults. To our knowledge, no study has examined the distinct impacts of guilt and shame proneness on both PTSD and DSO symptoms within this age group. This underscores the importance of incorporating individual differences in emotional style into trauma assessment and intervention.

Clinicians should therefore consider assessing and addressing these emotional tendencies in the treatment of trauma‐affected individuals, particularly in those with high levels of DSO or PTSD symptoms (Hyland et al. 2023). More specifically, therapeutic interventions such as EMDR or self‐compassion therapy may be a useful treatment target for PTSD and CPTSD, respectively, for symptoms of hyperarousal or avoidance and negative self‐concept and affect dysregulation (Rovaris et al. 2024; Karatzias et al. 2018). Interventions that explicitly target emotional regulation such as Enhanced Skills in Affective and Interpersonal Regulation (ESTAIR) may also be very useful for both the symptoms of PTSD and DSO (Karatzias et al. 2023, 2024).

Future research could explore the potential mediating role of additional emotional responses, such as anger or fear, in the trauma–PTSD/DSO relationship. Longitudinal studies could also provide further insight into the temporal dynamics of these relationships and whether interventions targeting guilt proneness and shame proneness reduce the long‐term risk of developing PTSD and DSO. It would also be interesting to determine if the direct and indirect effects reported in this study are moderated by other variables. For example, the type of trauma (e.g., sexual trauma) or overall level of psychological distress might be associated with stronger direct/indirect effects. The sample size precluded such analysis in this study.

While this study provides valuable insights, several limitations should be acknowledged. First, the cross‐sectional design limits the ability to draw causal inferences. Future research using longitudinal designs would help clarify the directionality of the relationships observed. Second, this study relied on self‐report measures, which may be subject to biases such as social desirability or memory recall bias. Participants' self‐reports regarding their trauma exposure might not capture the full scope or intensity of their experiences. Utilising a combination of self‐report and clinician‐administered assessments could provide a more comprehensive understanding of trauma's impact on emotional and psychological outcomes. Additionally, the outpatient group included individuals with different diagnoses and clinical histories. This heterogeneity represents a limitation, as emotional patterns and symptom severity may vary depending on the specific diagnosis and clinical condition of each participant. Future research could investigate how specific comorbidities (e.g., depression, anxiety disorders and borderline personality disorder) may interact with guilt and shame proneness to affect PTSD and DSO symptoms.

Another limitation of the present study is that the role of broader emotion regulation issues, such as emotional dysregulation, maladaptive coping strategies and emotional avoidance, in mediating the relationship between trauma exposure and PTSD/DSO symptoms was not extensively explored. These factors could provide further valuable insights into the underlying mechanisms that drive trauma‐related symptoms. Future research could build on our findings by investigating these emotional regulation processes in more depth to better understand how individuals manage their emotional responses after trauma and how these coping mechanisms might influence the development and persistence of PTSD and DSO symptoms.

In summary, this study underscores the significant roles of guilt and shame proneness in mediating the effects of total trauma exposure on PTSD and DSO symptoms. The findings suggest that emotional responses to trauma play a crucial role in the development of complex post‐traumatic psychopathology and highlight the importance of addressing these emotional responses in therapeutic settings. Further research is needed to explore additional mediators and to examine the long‐term impact of trauma‐related emotional distress.

## Conflicts of Interest

The authors declare no conflicts of interest.

## Supporting information


**Table S1.** Group descriptives and *t*‐test for all main study variables.

## Data Availability

Data are available upon reasonable request from the corresponding authors.
