# Supplementary material for: Shame and Guilt Proneness as Mediators of PTSD/DSO Symptoms in Young Adults
Source: Clin Psychol Psychother. 2025 Jul 29;32(4):e70131. doi: 10.1002/cpp.70131 (PMC12307099; doi:10.1002/cpp.70131)
Supplement: Supplementary file 1 — Table S1. Group descriptives and t‐test for all main study variables. [file CPP-32-e70131-s001.docx]

 Table S1. Group descriptives and t-test for all main study variables.

|  | | Group | | N | | Mean | | Median | | SD | | t (df) | p | Cohen’s d |
| --- | --- | --- | --- | --- | --- | --- | --- | --- | --- | --- | --- | --- | --- | --- |
| ITQ PTSD |  | Controls |  | 79 |  | 6.33 |  | 4.90 |  | 4.90 |  | -5.76 (155) | < .001 | -0.920 |
|  | | Outpatients |  | 78 |  | 11.6 |  | 6.44 |  | 6.44 |  |  |  |  |
| ITQ DSO |  | Controls |  | 79 |  | 6.29 |  | 4.17 |  | 4.17 |  | -6.17 (155) | < .001 | -0.986 |
|  | | Outpatients |  | 78 |  | 11.1 |  | 5.44 |  | 5.44 |  |  |  |  |
| ITQ Total Score |  | Controls |  | 79 |  | 12.62 |  | 7.97 |  | 7.97 |  | -6.64 (155) | < .001 | -1.060 |
|  | | Outpatients |  | 78 |  | 22.7 |  | 10.78 |  | 10.78 |  |  |  |  |
| PFQ Shame |  | Controls |  | 72 |  | 10.29 |  | 7.57 |  | 7.57 |  | -4.16 (150) | < .001 | -0.676 |
|  | | Outpatients |  | 80 |  | 15.6 |  | 8.07 |  | 8.07 |  |  |  |  |
| PFQ Guilt |  | Controls |  | 72 |  | 8.17 |  | 5.12 |  | 5.12 |  | -3.96 (150) | < .001 | -0.643 |
|  | | Outpatients |  | 80 |  | 11.7 |  | 5.88 |  | 5.88 |  |  |  |  |
| Total Trauma | | Controls |  | 80 |  | 3.46 |  | 3.00 |  | 2.45 |  | -2.52 (158) | < .05 | -.399 |
|  | | Outpatients |  | 80 |  | 4.53 |  | 4.00 |  | 2.86 |  |  |  |  |
|  | | | | | | | | | | | | | | |
